# Implementation of early essential neonatal care for newborns delivered by cesarean section in Jiaxing: a single-center prospective randomized controlled trial

**DOI:** 10.1186/s13006-024-00635-y

**Published:** 2024-05-03

**Authors:** Jianping Xu, Min Zhang, Yi Li, Shuiqin Gu

**Affiliations:** 1 Inpatient Preparation Center and Endoscopy Center, Jiaxing Maternity and Children Health Care Hospital, Affiliated Women and Children Hospital, Jiaxing University, Jiaxing, 314009 Zhejiang Province China; 2 Nursing Department, Jiaxing Maternity and Children Health Care Hospital, Affiliated Women and Children Hospital, Jiaxing University, No. 2468, Zhonghuan East Road, Nanhu District, Jiaxing City, 314009 Zhejiang Province China; 3 Jiaxing University Master Degree Cultivation Base, Zhejiang Chinese Medical University, Jiaxing University, No. 2468, Zhonghuan East Road, Nanhu District, Jiaxing City, Zhejiang Province 314009 China

**Keywords:** Doula company, Cesarean section, Early essential neonatal care, Maternal and child care, Breastfeeding

## Abstract

**Background:**

As an essential part of Early Essential Newborn Care, 90 minutes of mother-infant skin-to-skin contact is significant in improving maternal and infant outcomes. However, due to human resource constraints and the consideration of maternal and infant safety, it is difficult to achieve continuous uninterrupted skin-to-skin contact for at least 90 minutes during and after cesarean delivery. The aim of this study was to investigate the efficacy and safety of the continuous uninterrupted skin-to-skin contact for at least 90 minutes during and after cesarean section for exclusive breastfeeding rate during hospitalization and maternal and infant health indicators during and after cesarean delivery.

**Methods:**

This is a single-center, prospective randomized controlled trial conducted in one tertiary care hospital in China. We selected 280 cases of elective cesarean delivery in a tertiary maternal and child specialty hospital in Zhejiang Province from September 2018 to August 2022, which were randomly divided into two groups: in the conventional group, doulas performed at least 30 minutes for early continuous SSC within 10–30 minutes during and after cesarean delivery. In the EENC group, with immediate continuous SSC within 5–10 minutes of neonatal delivery until surgery is completed and continued SSC after returning to the ward. Exclusive breastfeeding rate during hospitalization and maternal and infant health indicators were compared between the groups.

**Results:**

A total of 258 cases were analyzed. Compared with the control group, the EENC group had earlier first breastfeeding initiation (13.7 ± 3.6 vs 62.8 ± 6.5 minutes, *P* < 0.001), longer duration of first breastfeeding (42.6 ± 9.0 vs 17.9 ± 7.5 minutes, *P* < 0.001), earlier onset of lactogenesis II (73.7 ± 3.6 vs 82.5 ± 7.4 hours, *P* < 0.001), higher breastfeeding self-efficacy score (128.6 ± 8.9 vs 104.4 ± 8.5, *P* < 0.001), higher Exclusive breastfeeding rate during hospitalization (88% vs 81%, *P* = 0.018), higher maternal satisfaction scores (18.9 ± 1.1 vs 14.0 ± 2.7, *P* < 0.001). Meanwhile the EENC group showed lower incidence of neonatal hypothermia (0% vs 4.6%, *P* = 0.014), lower neonatal hypoglycemia (0% vs 5.4%, *P* = 0.007) and less cumulative blood loss within 24 hours postpartum (254.2 ± 43.6 vs 282.8 ± 63.8 ml, *P* < 0.001).

**Conclusion:**

The implementation of EENC up to 90 minutes by caesarean doula company nurses is feasible and beneficial to maternal and infant health.

**Trial Registration:**

ChiCTR1800018195(2018-09-04).

## Background

The World Health Organization (WHO) recommends that mother-infant skin-to-skin contact (SSC) should begin with direct contact with the mother’s bare skin within 1 minute of birth [1]. Multiple randomized controlled studies have found the importance of cesarean SSC for breastfeeding [2–4]. CUI Xiaomei et al. [5] conducted a randomized controlled study on the effectiveness of SSC immediately after birth and SSC within 1 hour after birth on breastfeeding duration, initiation and duration of first breastfeeding, time to initiation of lactation II, breastfeeding self-efficacy scores, breastfeeding acceptance and satisfaction scores, and rates of exclusive breastfeeding at 24, 48, 72 hours and 42 days postpartum were significantly better in the spontaneous delivery immediate SSC group than that in the 30-min-of-birth initiation SSC control group. SSC within 1 minute of birth can be achieved during vaginal delivery. However, for caesarean delivery, due to the abdominal incision and the influence of the sterile area, intraoperative SSC is usually performed by positioning the newborn on the mother’s chest after the umbilical cord has been ligated, and it is usually initiated around 1–3 minutes after birth.

Although WHO defines SSC practice, existing research practices differ in the time of SSC initiation, duration, and mother and infant position [6], especially the variability and difficulty of SSC implementation in cesarean delivery. However, the recommendations for clinical implementation of SSC in cesarean delivery specify continuous SSC immediately after birth for at least 90 minutes cumulatively [7], and 90 minutes of immediate intraoperative health services is the best option, but human resources have been a barrier for SSC. Because a study abroad reported that a delivery guide during cesarean section could enhance safety and improve patient perception [8]. The recommendation of SSC immediately after birth for all women and their newborns, including cesarean delivery, is a core initiative of the EENC expert consensus [9], Subject to human resource constraints, during cesarean section, intraoperative initiation of uninterrupted continuous SSC for at least 90 minutes is a difficult task in performing EENC [10].

This study was based on a cesarean section with doula company nurse, which aimed to address the problem of immediate sustained intraoperative mother-infant skin-to-skin contact for 90 minutes, and an RCT was designed to validate the effectiveness. The timing of SSC initiation positively influenced breastfeeding and maternal and infant health indicators. Additionally, doulas should establish sufficient trust with the parturient to provide psychologic supportive care in the perioperative period and play an integral role in implementing EENC during cesarean section and postoperative EENC continuation care. 90 minutes mother-infant skin care during cesarean delivery performed by nurse guides is rare in the national and international literature. we conducted a randomized controlled study of immediate intraoperative continuous SSC for at least 90 minutes during cesarean delivery in China to provide an evidence-based basis for the promotion of EENC techniques in cesarean delivery.

## Methods

### Study population

According to the sample size calculation formula of the current survey, $${\displaystyle \begin{array}{l}n=\frac{{z^2}_{\textrm{a}}\times pq}{d^2}\\ {}\end{array}}$$, p is the expected EENC implementation rate; q = 1- p; d is the tolerance error, using d = 0.2p; and z is the standard normal distribution bound. The average rate of cesarean delivery EENC performed in Chinese hospitals is about 50%; α = 0.05, zα = 1.96; substitution into the formula yielded *n* = 96 cases, and the number of cases was expanded by a factor of 3 to 280 to account for a 20% loss rate. This prospective randomized controlled study included elective cesarean deliveries from September 2018 to August 2022 in a tertiary care hospital in Jiaxing City. The inclusion criteria were elective cesarean delivery; gestational week ≧37 weeks, combined spinal-epidural anesthesia, and those without language communication disorders and mental illness. Additionally, the exclusion criteria were pregnant women with serious pregnancy complications/comorbidities, contraindications to breastfeeding, intraoperative change of anesthesia, neonatal condition requiring immediate transfer to the neonatal intensive care unit (NICU), incomplete data, and unwillingness to cooperate. We selected 280 cases of elective cesarean delivery that were divided into the EENC and conventional groups by using the random number table method. The ethical review committee of Jiaxing Maternal and Child Health Hospital reviewed this project, and the informed consent form was signed.

## Research methodology

### Study overview

This study was a single-center prospective randomized (1:1 allocation) controlled trial. The pregnant women were randomly divided into EENC and conventional groups by using a random number table automatically generated by a computer. The staff who performed at least 90 minutes of SSC during and after cesarean were blinded to the randomization procedure. The study coordinator placed allocation details in a non-transparent, sealed envelope and concealed them from recruiters, data collectors, and allocators. After recruitment and baseline data collection, another study coordinator opened the envelopes and assigned participants to the EENC group and the conventional groups, which with the ending number is an odd number were in the EENC group and an even number of women were in the conventional group. Recruitment, data collection and data analysis were carried out by the corresponding assistant researchers, researchers and statisticians. Due to the nature of the study, participants were not blinded to the group’s intervention, but both physicians and Doulas were blinded.

### Establishment of an intraoperative EENC team for cesarean delivery

Doulas are trained professionals that provide comprehensive support during the cesarean delivery. Only full-time obstetric nurses with 3 years of experience, good communication skills, intraoperative EENC theory and skills training, and who passed the examination to ensure the homogenization of intraoperative SSC implementation steps were assigned to implement intraoperative EENC during cesarean delivery. Meanwhile, they were volunteered to perform intraoperative SSC as a doulas. Doulas in the two groups were identically qualified, which in the two groups helped the mother to make eyes contact with the newborn, and the routine nursing and psychological counseling were the same. Differing in mother-infant SSC**,** the EENC group, the skin contact between mother and newborn was carried out in strict accordance with the requirements of basic health care technology for newborns at early stage, and the skin contact lasted for 90 minutes immediately after the umbilical cord was cut off.

### Intervention methods

The following steps were performed in the conventional group: thorough drying of the newborn after delivery, delayed umbilical cord weaning, early mother–infant SSC (the guide nurse started SSC within 10–30 minutes after birth, and the duration of intraoperative SSC was at least 30 minutes, and the cessation of SSC at the latest postoperatively), and returning to the ward for routine care.

Moreover, the following steps were performed in the EENC group: After delivery of a newborn by cesarean section, the neonate was immediately placed supine on a dry towel on the mother’s abdomen, and drying of the neonate was started within 5 seconds. The drying maneuver was completed within 20 to 30 seconds and the newborn was thoroughly dried. During the drying of the newborn, the assistant touches the umbilical artery, waits for the umbilical artery pulsation to stop, and ties the umbilical cord approximately 1 to 3 minutes after birth. After ligating the umbilical cord, the operator hands the newborn to the guide nurse. Doulas initiated SSC within 5–10 minutes after birth until surgery is completed. At the end of the cesarean section, in order to ensure the safety of the newborn, the newborn was temporarily separated from the mother, and when the mother was moved to the surgical cart postoperatively, the newborn was placed on the mother’s chest to continue the SSC was continued, returning to the ward to continue SSC to accumulate ≧ 90 minutes, reaching the time when the newborn can stop breastfeeding on their own, and after-care routine similar to the conventional group.

Intraoperative immediate SSC safety management points include the correct procedure of performing chest–skin warming; the newborn should be in a prone position on their mother’s bare chest after cutting the umbilical cord; the newborn’s chest and abdomen should touch their mother’s chest for maximum skin contact; they should face toward the breast; pay attention to warmth; when the newborn starts using their tongue, the head should be turned or lifted, and other breastfeeding signals should be performed to help complete the first breastfeeding; intraoperative SSC should continue until surgery is completed. Meanwhile, doulas should always observe the newborn’s skin color, breathing, sucking response and the routine check-up and weighing should be recorded and completed before returning to the ward.

### Observed indicators

1. *Breastfeeding indicators:* ① Breastfeeding initiation and the duration of first breastfeeding: initiation of direct skin contact of the mother’s chest and abdomen against the newborn’s chest and abdomen skin contact after delivery of the newborn [11], the newborn correctly latches the nipple and most of the areola, and the establishment of regular effective sucking and swallowing [12] for the start of breastfeeding time and duration. ② Onset of lactogenesis II: the time point approximately 72 hours after delivery when large quantities of breast milk starts to be secreted, at which time the mother perceives that the milk rises and the breast is full [13]. Timing of initiation of initiation of lactation phase II by a combination of maternal report and staff assessment. Staff will informed the phenomenon of the onset of lactogenesis II. to mother advancely. And around 48–72 hours postpartum, most women feel that their breasts were fullness or swelling and their breasts products abundant milk. When they felt the above-mentioned situation, they should informed the staff immediately. To ascertain the onset of the lactogenesis II. staff will observe and squeeze both sides of the areola,and then assess milk spillage. ③ Breastfeeding self-efficacy score: the breastfeeding self-efficacy scale (BSES) was scored [14] between 30 and 150, with higher scores representing higher breastfeeding self-efficacy and higher self-confidence in independent breastfeeding skills. ④ Exclusive breastfeeding rate during hospitalization: newborns were exclusively breastfed, except for administration of vitamins and minerals. The rate of exclusive breastfeeding for mothers who underwent cesarean delivery during hospitalization was calculated for both groups. ⑤ Maternal satisfaction scores: a self-designed cesarean delivery satisfaction questionnaire was distributed before discharge, including 10 items (cesarean delivery maternal and infant care, first maternal and infant SSC, breastfeeding, maternal and infant cesarean delivery outcome), with each item scored on a Likert 5-point scale (total score of 5 to 20), with higher scores indicating higher maternal perceptions. The questionnaire’s Cronbach alpha coefficient was 0.839 after the pre-survey.
2. *Maternal and infant health indicators:* ① Incidence of neonatal hypothermia: the axillary temperature of the newborn is < 36.0 °C at any time within 24 hours after birth. ② Incidence of neonatal hypoglycemia: neonatal heel blood glucose of < 2.2 mmol/L at any time within 24 hours after birth. We only monitor blood glucose in newborns at high risk for hypoglycemia. ③ Cumulative blood loss within 24 hours postpartum:it was measured by weighing the weight of the immersion pad and then minus the weight of the dry pad. The weight of 100 g is about equal to 100 ml(ml) of blood.

## Statistical methods

SPSS 25.0 software was used for the statistical analysis of the collected data. Measurement data were analyzed using x ± s, and t-test or Fisher test was used to compare two independent samples between groups. The count data were presented as frequency and rate, and χ^2^ test was used for comparison between groups.

## Results

**Fig. 1:**
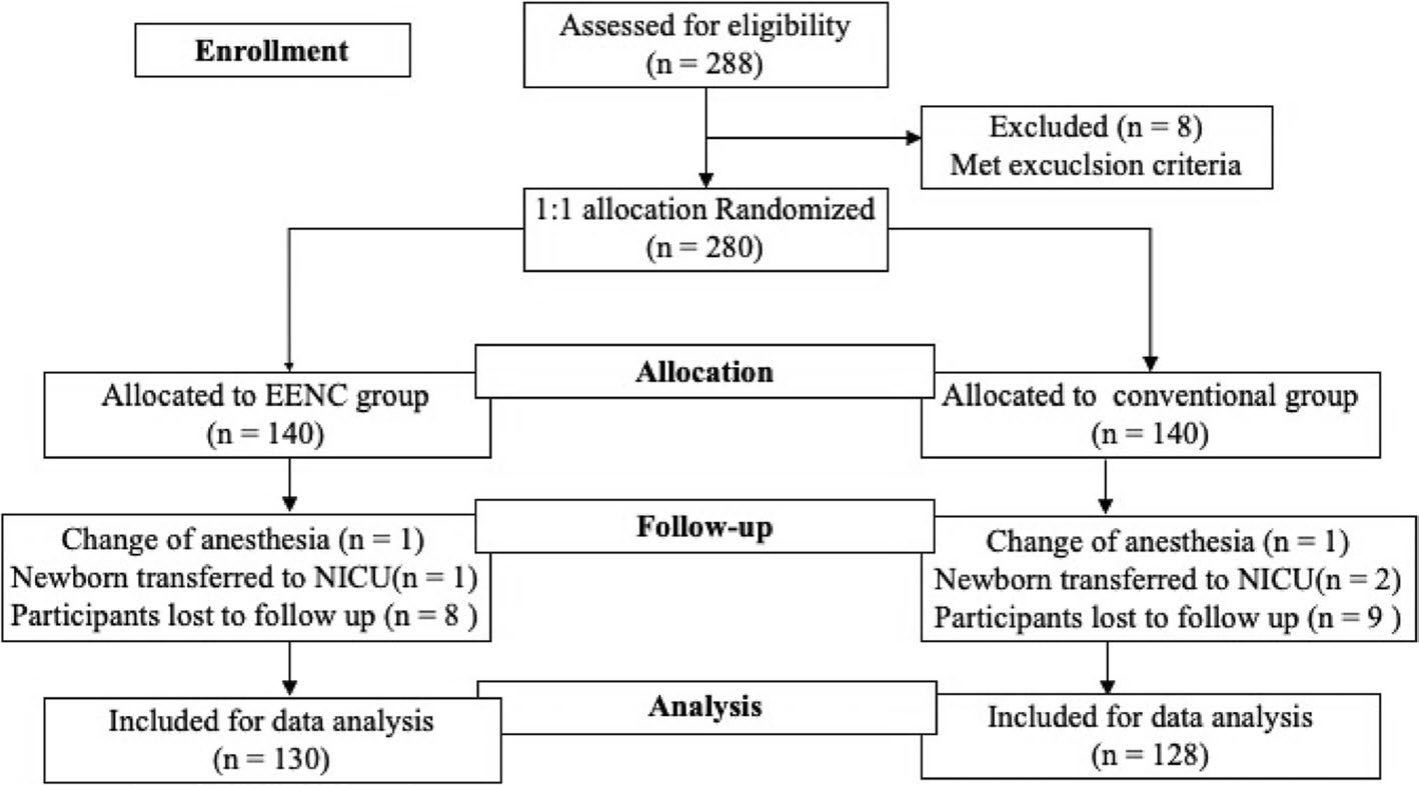
Flow chart of the study

In total, 280 pregnant women with elective cesarean delivery were included in this study from September 2018 to August 2022 and they were randomly allocated to EENC group and conventional group. Data were completed for 130 women in the EENC group and 128 in the conventional group. During the study, 2 cases in the EENC group dropped out because of the change of anesthesia model, and the immediate baby transfer to NICU due to neonatal condition respectively, and 8 cases were dropped out because of the participants were lost to follow up. In conventional group, one case dropped out because of the intraoperative change of anesthesia model, 2 cases dropped out because of the immediate baby transfer to NICU due to neonatal condition, and 9 cases dropped out because of participants were lost to follow up. Finally 130 in the EENC group and 128 in the conventional group were analized (Fig. 1).

There were 100 (77%) primigravidas in EENC group and 96 (75%) primigravidas in conventional group. The average age of the EENC and conventional groups was 30.6 ± 4.1 and 29.8 ± 4.5 years, respectively. There were no significant differences found between groups in maternal age, gestational week, newborn birth weight and Apgar scores before intervention, as shown in Table 1. Table 2 shows the comparison of breastfeeding indicators. Breastfeeding initiation and the duration of first breastfeeding, onset of lactogenesis II, breastfeeding self-efficacy score, exclusive breastfeeding rate during hospitalization and maternal satisfaction scores improved in the EENC group compared with the conventional group, with statistically significant differences.

**Table 1:** Comparison of baseline information of mothers and infants in the two groups (^−^*x ± s*)

Item	EENC Group (*n* = 130)	Conventional Group (*n* = 128)	*t* / *χ2*	*P*
Maternal age ($\overline{x}$ ±s,age)	30.6 ± 4.1	29.8 ± 4.5	1.327	0.186
Primigravida Case (%)	42(32)	37(29)	0. 351	0.553
College and above [Case (%)]	100(77)	96(75)	0.131	0.718
Gestational Week ($\overline{x}$ ±s,w)	38.8 ± 1.0	38.8 ± 1.0	0.092	0.927
Newborn birth weight ($\overline{x}$ ±s,g)	3340 ± 358	3293 ± 366	1.019	0.309
Maternal hemoglobin (g/l)	122.1 ± 6.7	122.2 ± 6.8	0.085	0.932
Apgar score	9.0 ± 0.5	9.0 ± 0.4	0.513	0.609

**Table 2:** Comparison of breastfeeding-related indicators between the two groups ($\overline{x}$ *±s*)

Item	EENC Group (*n* = 130)	Conventional Group (*n* = 128)	t	*P*
Breastfeeding initiation (min)	13.7 ± 3.6	62.8 ± 6.5	73.193	< 0.001
Duration of first breastfeeding (min)	42.6 ± 9.0	17.9 ± 7.5	23.938	< 0.001
Onset of lactogenesis II(h)	73.7 ± 3.6	82.5 ± 7.4	12.145	< 0.001
Breastfeeding Self Efficacy Scale (points)	128.6 ± 8.9	104.4 ± 8.5	22.206	< 0.001
Exclusive breastfeeding rate during hospitalization (%)	226(88)	210(81)	5.556	0.018
maternal satisfaction scores (points)	18.9 ± 1.1	14.0 ± 2.7	18.593	< 0.001

Table 3 shows the maternal and infant health indicators. In total, there were significant differences in the incidence of neonatal hypothermia, neonatal hypoglycemia, and cumulative blood loss within 24 hours postpartum between the two groups (*P* < 0.05). The cumulative blood loss within 24 hours postpartum in the EENC group was significantly lower than that in the conventional group after at least 90 minutes intervention (254.2 ± 43.6 mmol/L VS 282.2 ± 63.8 ml，*P* < 0.001). Compared with the conventional group, a higher number of newborns experienced neonatal hypothermia (4.6% vs 0%, *P* = 0.014) and neonatal hypoglycemia (5.4% vs 0%, *P* = 0.007) in EENC group.

**Table 3:** Comparison of maternal and infant health indicators between the two groups [cases (%)]

Item	EENC Group (*n* = 130)	Conventional Group (*n* = 128)	T/Fisher	*P*
Incidence of neonatal hypothermia (person/%)	0(0)	6(4.6)	6.215	0.014
Incidence of neonatal hypoglycemia (person/%)	0(0)	7(5.4)	7.729	0.007
Cumulative blood loss within 24 hours postpartum (ml)	254.2 ± 43.6	282.8 ± 63.8	4.206	< 0.001

## Discussion

### Analysis of the role of immediate intraoperative SSC for at least 90 minutes in improving breastfeeding during cesarean delivery

Immediate SSC creates conditions for the first intraoperative establishment of suckling by the infant, and earlier suckling of the nipple increases lactogen receptors in the breast [15] and promotes breastfeeding. A cross-sectional study of the association between EENC and breastfeeding in eight countries in Asia and the Pacific reported that newborns should receive immediate SSC without interruption for at least 90 minutes to maximize early and exclusive breastfeeding, regardless of the delivery mode [16], confirming the importance of the timeliness of SSC. Moreover, another multicenter study reported a strong dose–response relationship between early SSC duration and inpatient exclusive breastfeeding [17]. In this study, immediate and continuous SSC within 8 minutes after delivery, and initiation of the first breastfeeding within 20 minutes after birth when the neonate is more alert, which was cumulatively longer than the conventional group, promoted early Onset of lactogenesis II. Immediate initiation of SSC increased the rate of exclusive breastfeeding during hospitalization from 43 to 73.4%, and the duration of first breastfeeding increased from 15.8 to 17.1 minutes [18]. In this study, the primary outcome also showed the significant differences in EENC group,which is better than conventional group. EENC group’s breastfeeding indicators such as breastfeeding initiation and the duration of first breastfeeding, the onset of lactogenesis II, breastfeeding self-efficacy score, exclusive breastfeeding rate during hospitalization and maternal satisfaction scores were all better than conventional group.

### Safety and human resource analysis of doulas on immediate SSC during cesarean delivery

Although studies showed the benefits of cesarean EENC [19] and improved outcomes for mothers and infants, and reduced labor hazards with the continuous support from a guide nurse during spontaneous delivery [20], immediate intraoperative continuous SSC during cesarean delivery has risk factors, such as neonatal asphyxia, bed fall, and maternal chest distress. The obstetric guide nurse acts as a dedicated EENC nurse to meet the key factors of maternal intraoperative needs while providing intraoperative psychological support, one-on-one support by professionals, immediate SSC, and breastfeeding guidance. Although EENC was introduced in China in 2016, spontaneous delivery was promoted recently in pilot cities in implementing EENC, and the SSC program, especially the intraoperative promotion of cesarean EENC, has encountered hindrances. The current intraoperative SSC practice in cesarean delivery is rarely reported, and most of the reports focused on human resources, cesarean incision infection, postoperative pain, and other factors that limit the early continuation of intra- and postoperative SSC. Thus, we selected obstetric nurses as doulas to assist in immediate intraoperative SSC during cesarean delivery, and they efficiently bridged the continuity of pre-, intra-, and postoperative EENC as a doulas companion. The maternal perception of cesarean delivery was rated much higher in the EENC group than in the conventional group, and the difference was statistically significant. Because the patients experienced strong support from the hospital management, and the implementation of the program does not pose risks to mothers and infants, the incidence of neonatal hypothermia, hypoglycemia, and postpartum hemorrhage has improved. However, the implementation process did not actively withdraw due to maternal factors, and the safety and feasibility was confirmed.

## Conclusions and limitations

The three core measures for implementing EENC during cesarean section are thorough drying, delayed umbilical weaning, and SSC. However, difficulty in clinical promotion may be due to the duration of at least 90 minutes of immediate continuous SSC, and researchers have suggested that the new guidelines should include initiating SSC in the operating room for at least 15 minutes during the golden hour of the new life after cesarean section [21]. The unfamiliar environment of the operating room makes mothers nervous and anxious, and the doulas should educate the pregnant women regarding EENC preoperatively, establish a good nurse–patient relationship during hospitalization, and accompany and implement EENC throughout the cesarean section. Maternal trust and gratitude to the guide nurse would enhance the effectiveness of intraoperative SSC [22], but no study has investigated the effect of SSC on postpartum depression. A study showed that immediate intraoperative continuous SSC during cesarean delivery resulted in a positive maternal experience [23]. Another multicenter study on the impact of SSC initiation and duration on breastfeeding before and after implementing EENC changed the clinical practice of natural childbirth [24], but the focus on maternal needs and choices is limited both nationally and internationally. The present study is a single-center study with limitations, and a multicenter, double-blind, multi-group comparative study of EENC for cesarean delivery with a focus on long-term maternal and infant psycho-behavioral outcomes in multiple hospitals should be conducted in the future.

## Data Availability

The datasets used in the current study are available from the corresponding author upon reasonable request.

## Abbreviations

EENC: Early essential neonatal care
WHO: World health organization
SSC: Skin-to-skin contact
BSES: Breastfeeding self-efficacy scale
NICU: Neonatal intensive care unit
